# Investigation and validation of labelling *loop mediated isothermal amplification* (LAMP) products with different nucleotide modifications for various downstream analysis

**DOI:** 10.1038/s41598-022-11320-7

**Published:** 2022-05-03

**Authors:** Christian Warmt, Ceren Yaslanmaz, Jörg Henkel

**Affiliations:** Fraunhofer Institute for Cell Therapy and Immunology - Bioanalytics and Bioprocesses (IZI-BB), 14476 Potsdam, Germany

**Keywords:** Biochemical assays, Analytical biochemistry, Lab-on-a-chip, Biological techniques, Microbiology techniques, Chemical modification, DNA, Biochemistry, RNA

## Abstract

Loop mediated isothermal amplification (LAMP) is one of the best known and most popular isothermal amplification methods. It's simplicity and speed make the method particularly suitable for point-of-care diagnostics. Nevertheless, false positive results remain a major drawback. Many (downstream) applications are known for the detection of LAMP amplicons like colorimetric assays, in-situ LAMP or CRISPR-Cas systems. Often, modifications of the LAMP products are necessary for different detection applications such as lateral flow assays. This is usually achieved with pre-modified primer. The aim of this study is to evaluate amplicon labelling with different modified nucleotides such as Cy5-dUTP, biotin-dUTP and aminoallyl-dUTP as an alternative to pre-labelled primers. To realise this, the effects on amplification and labelling efficiency were studied as a function of molecule size and nucleotide amount as well as target concentration. This research shows that diverse labelling of LAMP amplicons can be achieved using different, modified nucleotides during LAMP and that these samples can be analysed by a wide range of downstream applications such as fluorescence spectroscopy, gel electrophoresis, microarrays and lateral flow systems. Furthermore, microarray-based detection and the ability to identify and distinguish false positives were demonstrated as proof of concept.

## Introduction

In 2000, Notomi et al. developed a novel isothermal amplification technology named *loop mediated isothermal amplification* (LAMP)^1^. More than two decades later, LAMP belongs to the most popular isothermal amplification techniques^2^. Based on a specialized set of up to 6 primer and a strand displacement polymerase^3^, this method enables a highly sensitive and specific amplification of low amounts of target DNA^4^. In combination with a reverse transcriptase it's also suitable for RNA amplification (RT-LAMP)^5^. Until now, more than thousand publications using this technology in many variations like RT-LAMP^6^, RIME-LAMP^7^ or DARQ-LAMP^8^ can be found. Since the beginning of the COVID-19 pandemic, more than 800 publications have been published on the detection of SARS-CoV-2. Of these, about 100 deal with LAMP as a detection method. This emphasizes the importance of the LAMP not only for research in general but also for current important issues worldwide.

Amplification and detection of nucleic acid using LAMP has many advantages compared to none isothermal, PCR-based methods. In first place, there is the amplification speed. By using an appropriate primer set and optimal amplification conditions, it is possible to detect low amounts of target nucleic acid in less than 30 min^9–11^, compared to amplification times of up to 3 h for the PCR process^12^. Furthermore, an integration and usage of LAMP in point-of-care devices is highly recommended because of the reduced demand of energy and technical equipment^13,14^. Despite many LAMP advantages, false positives during amplification caused by contamination, sequence similarities and primer dimers are well known LAMP disadvantages^15,16^.

There is a broad range of combination possibilities of LAMP with downstream analytical systems for the detection of amplification products. They include standard gel electrophoresis^17^ and quantitative amplification (qLAMP)^18^ over different colorimetric reagents (phenol red^19^, leuco crystal violet^20^, SYBR green^21^, malachite green^22^) to lateral flow assays (LFA)^23^ or combinations with CRISPR-Cas Systems^24^. The most commonly used are LFA and colorimetric assays but the major limitation of the mentioned techniques is that they are not suitable for the distinction of false positive results. Microarray based detection methods could help to overcome this hurdle.

In this process, specific, single-stranded DNA probes with a length of about 20–30 nt are immobilised on a modified surface. Following amplification, the products can hybridize highly specific on these probes. With a suitable selection of probes, it is possible to distinguish the amplicons not only from false positives, but even from single nucleotide polymorphisms (SNPs). Following this hybridization, the signals of the labelled and hybridized amplicons are read out^25,26^.

Unfortunately, recent literature does not suggest any microarray based detection method in combination with LAMP solving the “false positive problem”.

The use of (semi)quantitative analysis systems (e.g. qLAMP) and lateral flow based methods require labelling of amplicons for target detection. So far, using pre-labelled primer or probes has been the method of choice^8,27,28^. Reduced flexibility and high costs associated with huge primer sets, inflexible adjustment of signal intensity or amplification efficiency may, but do not necessarily, be a consequence of this.

Alternatively, target labelling can be performed during amplification with pre-labelled nucleotides. These types of nucleotides are available with different modifications such as fluorophores, biotin or amino functionalization for subsequent custom labelling.

In a previous study we demonstrated that this labelling is useful for isothermal methods such as *recombinase polymerase amplification* (RPA) for a sensitive detection of pathogens and differentiation of single nucleotide polymorphism (SNP) gene variants^26^. Therefore, we were highly motivated to investigate the suitability of this labelling technique for other isothermal methods. Only a handful of examples exist that use modified nucleotides for direct LAMP labelling and are mostly combined with in-situ LAMP^23,29^. In a previous study, we demonstrated the incorporation of biotin-labeled dUTP in combination with (semi)quantitative lateral flow analysis^23^.

This study aims to analyse and assess the described direct and flexible LAMP based labelling method. Moreover, it extends and refines our analysis and demonstrates the advantages and disadvantages of labelling LAMP amplicons with 5-(3-Aminoallyl)-2'-deoxyuridine-5'-triphosphate (Cy5-dUTP), γ-[N-(biotin-6-amino-hexanoyl)]-(5-aminoallyl)-2'-deoxyuridine-5'-triphosphate (biotin-dUTP) and 5-aminoallyl-dUTP (AA-dUTP) representative for small, medium and large size nucleotide modifications.

**Figure 1:**
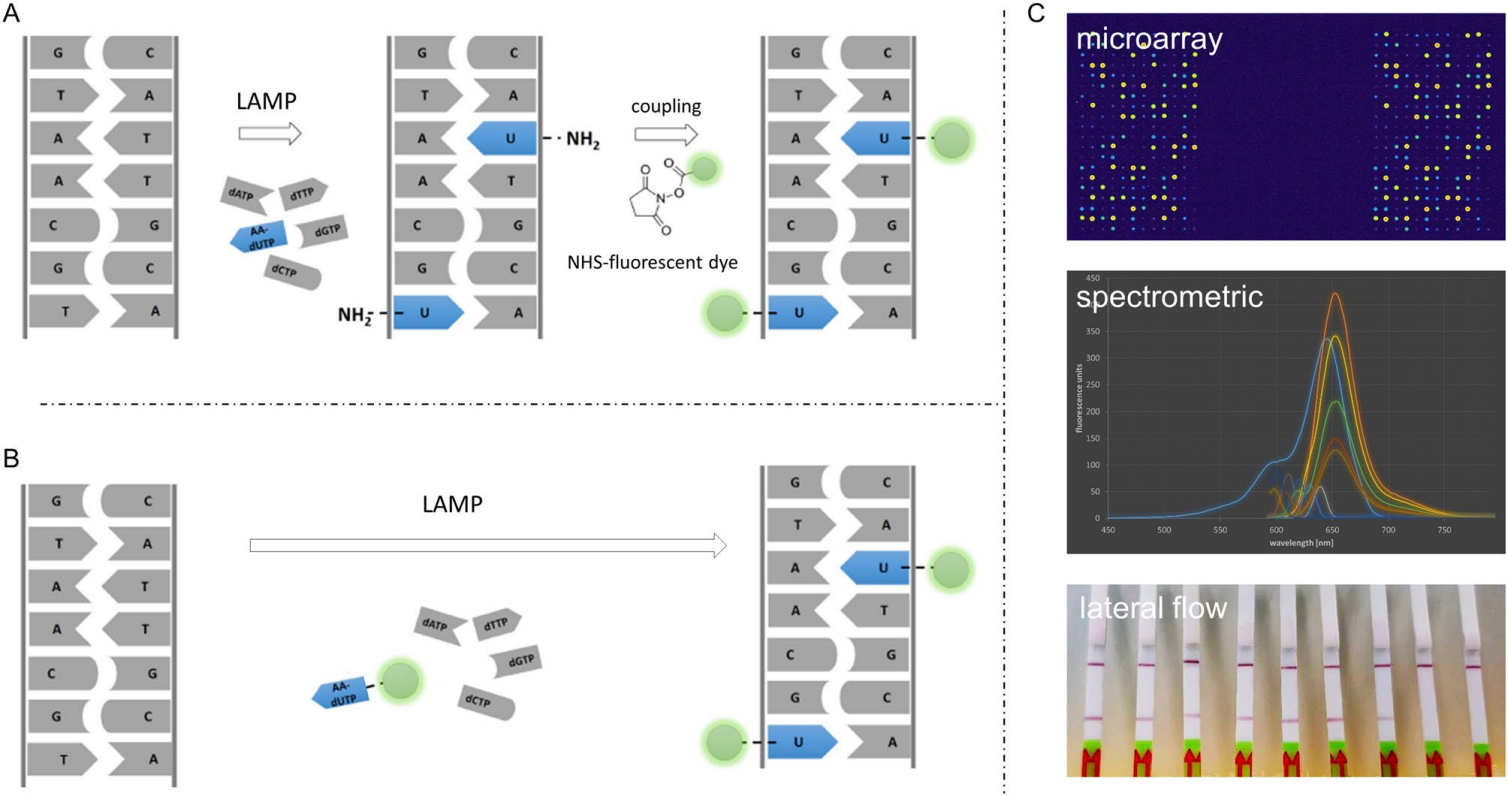
LAMP dUTP-labelling strategies. In this study, direct and indirect labelling of LAMP amplicons is shown. (**A**) Via indirect labelling, 5-aminoallyl-dUTP was introduced into the LAMP products and Cyanine-5 NHS-ester was coupled in a subsequent reaction. (**B**) Using direct labelling, modifications were incorporated into LAMP products using Cy5-dUTP and biotin-dUTP without additional reactions. (**C**) Amplicons labelled directly or indirectly via modified nucleotides can be used in various applications like microarray technology, spectroscopic analysis or lateral flow based systems.

The aforementioned nucleotide modifications not only have different molecular sizes, but also are useful in different assay and detection systems (Fig. 1). While Cy5-dUTP can be used for direct labelling of amplicons with fluorophores for a wide range of fluorescence detection methods, incorporated biotin-dUTP enables non-covalent immobilisation on streptavidin-modified surfaces in the commonly used LFA. AA-dUTP, on the other hand, is thought to allow the incorporation of a functional group that may be useful for subsequent labelling with NHS molecules.

In this study labelling and amplification efficiency using different amounts of x-labelled nucleotides and SARS-CoV-2 cDNA during amplification were compared performing qLAMP and spectroscopic analysis.

Various detection methods were used to show a broad range of possibilities for downstream analysis like fluorescence spectroscopy analysis and microarray technology as well as lateral flow based detection.

Due to a lack of literature on microarray based LAMP detection for the distinction of false positives, this work will be considered as proof of concept study.

For this reason, labelling of *Salmonella* and *E. coli* DNA with Cy5-dUTP was performed and LAMP was coupled with a subsequent microarray analysis to enable the identification of false positive results.

## Results

### Effect of x-dUTP incorporation on LAMP efficiency

**Figure 2:**
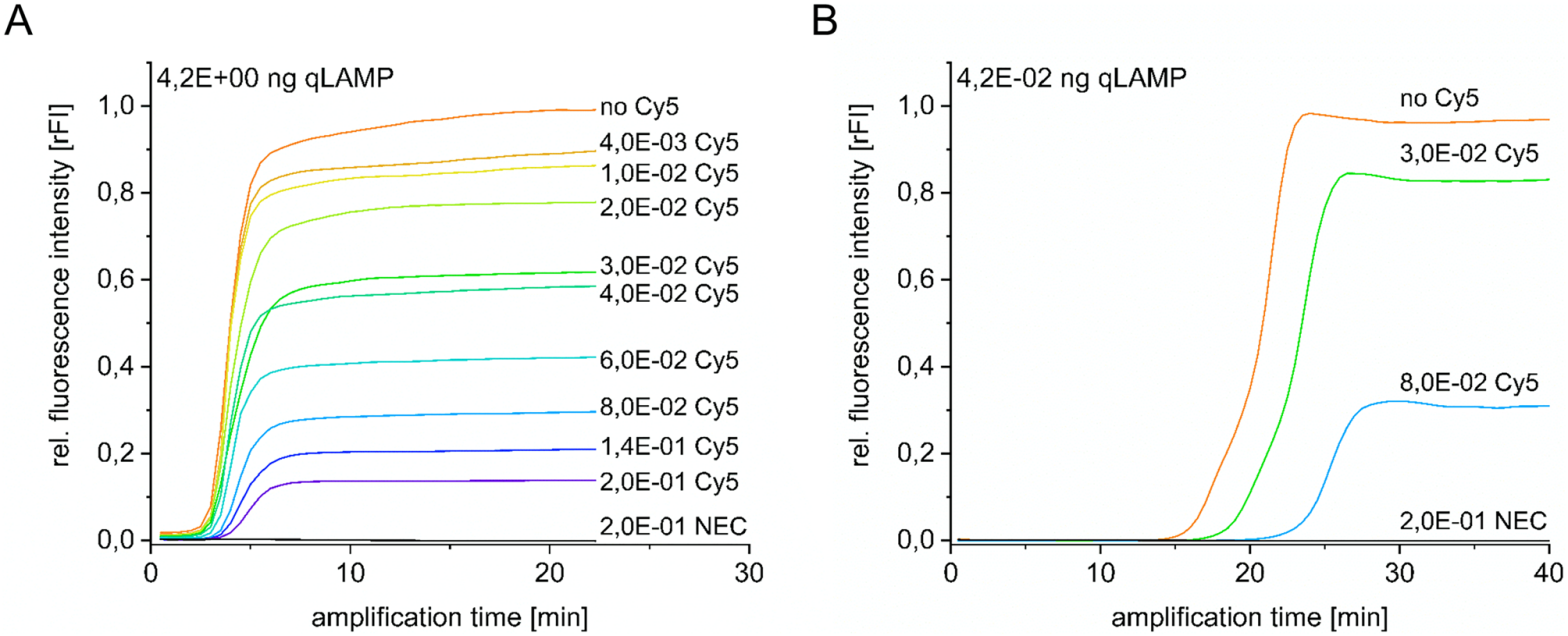
Influence of labelled nucleotides on amplification efficiency. Presented are SYBR green based qLAMP results for the observation of amplification efficiencies using varying Cy5-dTUP and DNA concentrations per LAMP reaction. Relative fluorescence intensity (rFI) corresponds to SYBR green used for qLAMP analysis and is set to the highest fluorescence value reached by positive control (no Cy5). (**A**) shows a Cy5-dUTP dilution series from 2.0E-01 mM to 4.0E-03 mM using constant DNA amounts of 4.2E + 00 ng cDNA including a positive control (no Cy5) and a no enzyme control (NEC). (**B**) shows representative amplification curves for three different Cy5-dUTP amounts (no Cy5; 3.0E-02 mM and 8.0E-02 mM) using lower start DNA concentrations for visualisation of Ct-value shift caused by Cy5 labelled nucleotides.

To determine the effect on amplification efficiency by using labelled nucleotides during LAMP, SYBR green based quantitative *loop mediated isothermal amplification* (qLAMP) with various amounts of SARS-CoV-2 cDNA and 5-(3-aminoallyl)-2'-deoxyuridine-5'-triphosphate (Cy5-dUTP) as well as biotin-X-(5-aminoallyl)-dUTP (biotin-dUTP) and 5-aminoallyl-dUTP (AA-dUTP) dilution series was performed. With cDNA amounts of 4.2E + 00 ng per reaction and Cy5-dUTP concentrations ranging from 4.0E-03 mM to 2.0E-01 mM, target DNA has been detected in less than 5 min (Fig. 2). Sigmoidal like curves could be observed with SYBR green relative fluorescence intensities (rFI) ranging from 1.0 rFI for reactions without Cy5-dUTP and 0.93 rFI using 4.0E-03 mM Cy5-dUTP to 0.15 rFI for the highest Cy5-dUTP concentration (2.0E-01 mM) per reaction (Fig. 2A). There is only a slight difference in reaction efficiency using this amount of target DNA, as mirrored by the qLAMP cycle threshold (Ct) ranging from 2.96 min for the positive control (no Cy5-dUTP) and 3.07 min using 4.0E-03 mM Cy5-dUTP to 5.03 min when using 2.0E-01 mM labelled nucleotides. Maximum fluorescence intensity as well as Ct values decreased or increased in the order of labelling molecules per LAMP reaction.

Nevertheless, a decrease in amplification efficiency has been observed when using lower amounts of target DNA. The addition of only 4.2E-02 ng cDNA per LAMP reaction increased the overall Ct-values (Fig. 2B). While a Ct difference of 2.96 min occurred when high amounts of DNA were used, a Ct-value shift of more than 13.00 min from the lowest to the highest amount of Cy5-dUTP per reaction in diluted samples was observed. A no enzyme control (NEC) was used as negative control with maximum amounts of Cy5-dUTP and showed no amplification curves.

**Figure 3:**
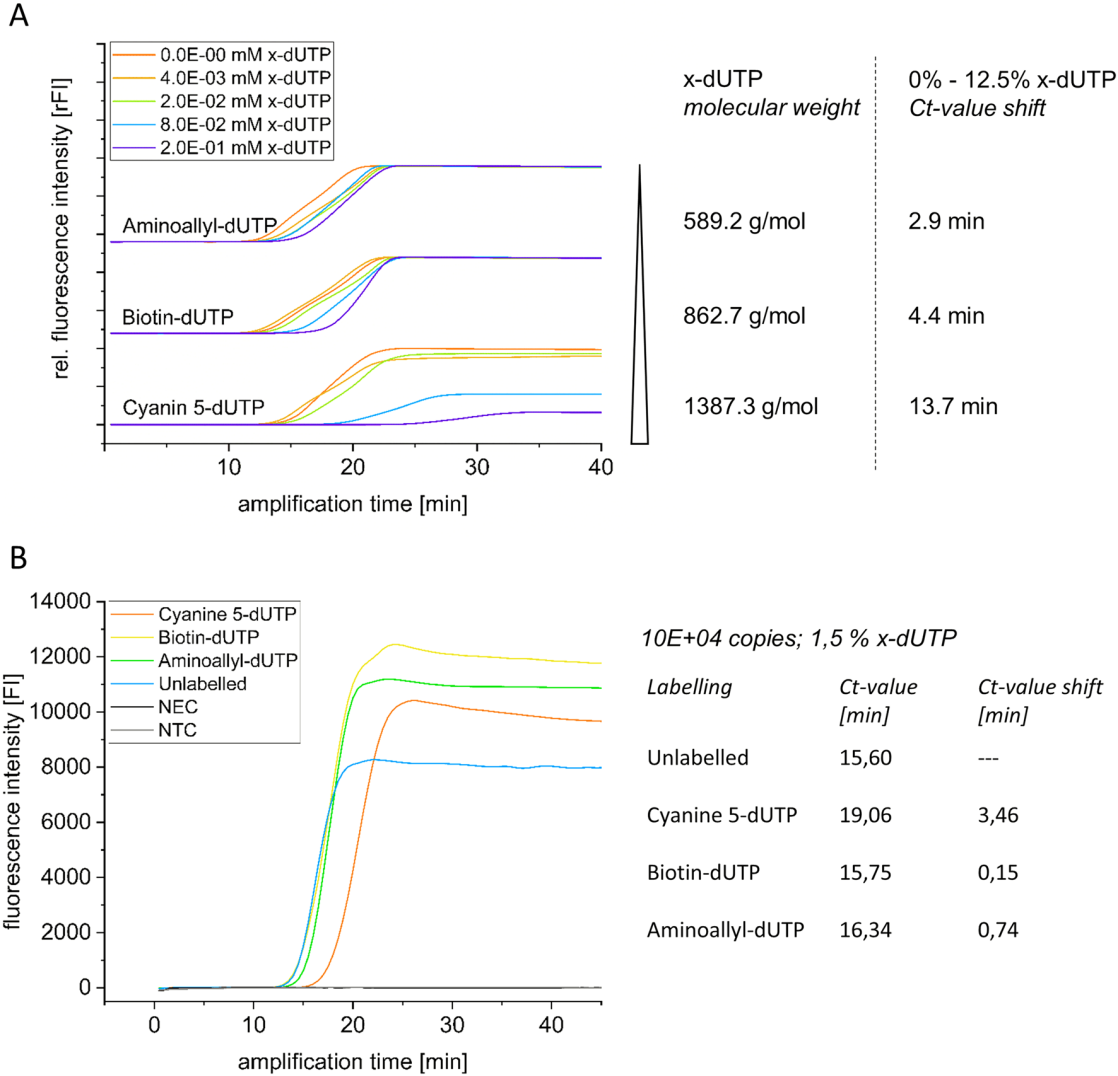
Influence of the molecule size and concentration. (**A**: left) Shown are SYBR green based qLAMP curves for amplification of 4.2E-02 ng target DNA using various labelled dUTPs with different molecular sizes for the incorporation into the amplicons. All x-dUTP variants were tested in a range from 4.0E-03 mM to 2.0E-01 mM per LAMP reaction that corresponds to 0.3%, 1.4%, 5.4% and 12.5% x-dUTP compared to the overall dUTP + dTTP amount. (**A**: right) Molecular weights corresponding to the three nucleotide modifications and Ct-value shifts from lowest to highest concentration are depicted. (**B**: left) Shown are SYBR green-based qLAMP curves for amplification of approximately 10^4^ copies of a SARS-CoV-2 cDNA with 1.5% labelled nucleotides (NEC: no enzyme control, unlabelled; NTC: no template control, unlabelled). (**B**: right) Ct-values and corresponding Ct-value shifts performing amplification with unlabelled and x-dUTP-labelled nucleotides.

Since Cy5-dUTP is a comparatively large molecule in contrast to unlabelled nucleotides, we investigated the effects on amplification efficiency using various dUTP labels with different molecule sizes. For this AA-dUTP (589.2 g/mol), biotin-dUTP (862.7 g/mol) and Cy5-dUTP (1387.3 g/mol) were used with different amounts ranging from 0.28% to 12.5% (4.0E-03 mM to 2.0–01 mM) compared to the overall x-dUTP + dTTP amounts (Fig. 3).

In the tested range of x-dUTP endpoint amplification could be observed in less than 22 min, except for the 8.0E−02 mM and 2.0E−01 mM Cy5-dUTP samples. The use of the above mentioned amount of fluorescent-labelled nucleotides results in an increase of the enpoint amplification time to up to 27 min and 35 min, respectively. A clear correlation was found between the molecule size, represented as molecular weight, and the required amplification time. While using AA-dUTP, only slight qLAMP Ct-value shifts of less than 3 min from zero percent to 12.5% AA-dUTP per LAMP reaction are detected. This value increases to 4.4 min when using the slightly larger biotin-dUTP and is more than 13 min using Cy5-dUTP.

To investigate the influence of labelling on the amplification under realistic assay conditions, 10E + 04 copies of the SARS-CoV-2 cDNA fragment were amplified with 1.5% x-dUTP/98.5% dTTP each. Successful and complete endpoint amplification was observed for all samples within 25 min (Fig. 3B). The unlabelled reference sample showed a Ct-value of 15.6 min. Labelling with biotin-dUTP and AA-dUTP also resulted in Ct-values of less than 16.5 min. The use of Cy5-dUTP influenced the amplification time the most, with a Ct-value shift of 3.5 min.

### Cy5-dUTP incorporation and -analysis

**Figure 4:**
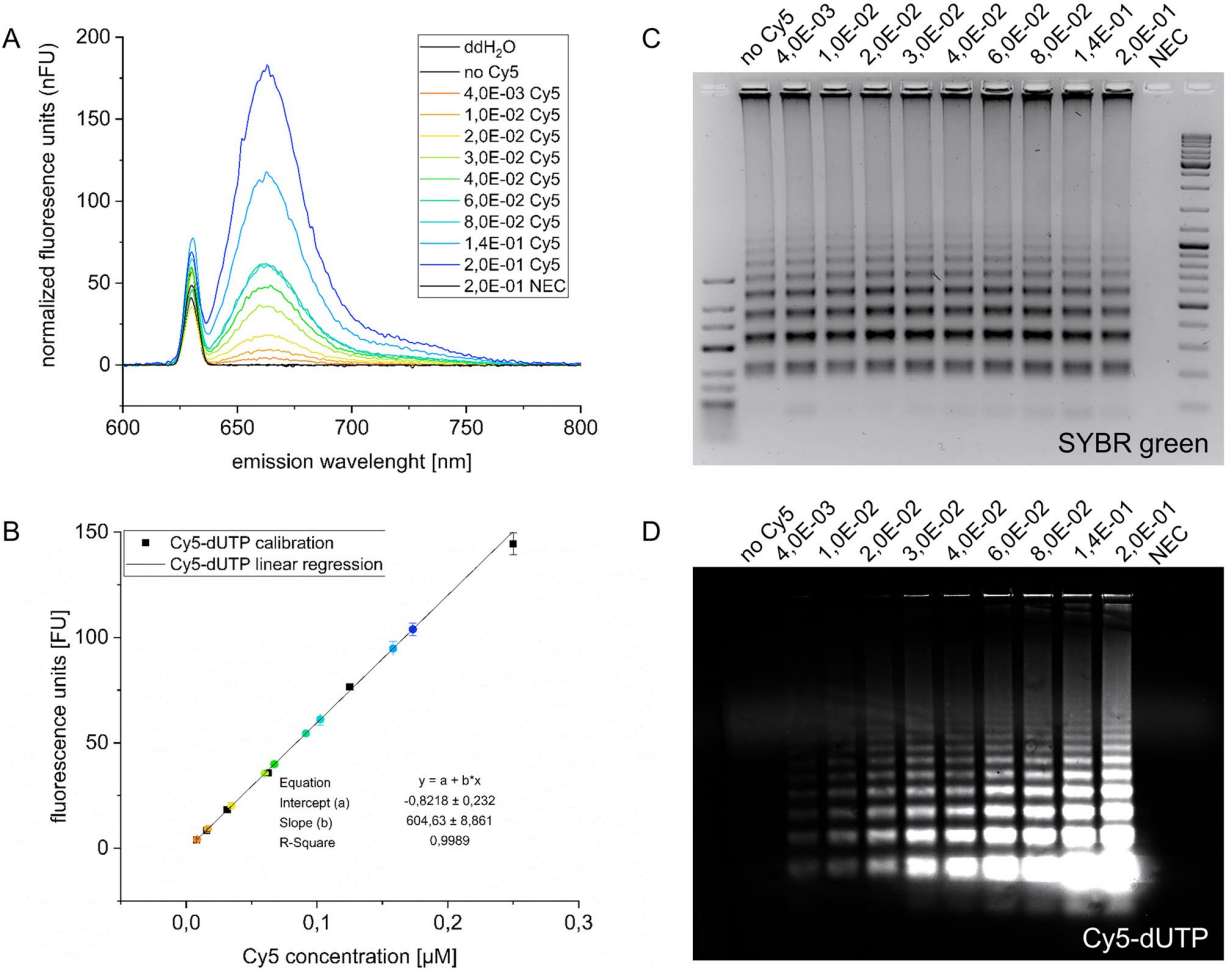
Detection of Cy5-dUTP in LAMP amplicons. Incorporation of fluorescent-labelled nucleotides was analysed by fluorescent spectroscopy as well as fluorescent gel electrophoresis. (**A**) Fluorescent spectra with Cy5-dUTP dilution series used in endpoint LAMP (4.0E-03 mM to 2.0E-01 mM). NEC and no-Cy5 representing LAMP positive and negative control. Fluorescent intensities are normalized to a uniform amplicon concentration measured after purification of LAMP products. (**B**) Calibration line (black dots and line) for calculation of incorporated Cy5 molecules for all sample (coloured dots). Colour scheme is the same as shown in (**A**). (**C**) Common SYBR green based gel electrophoresis with samples shown in (**A**). (**D**) Same gel as shown in (**C**) recorded by a microarray scanner to detect Cy5 fluorescent LAMP products.

The rate of Cy5 incorporation into LAMP amplicons was calculated using direct labelling via Cy5-dUTP and an indirect method via AA-dUTP with subsequent NHS-Cy5 labelling. To overcome inconsistent results by uncompleted LAMP reactions an endpoint LAMP (60 min) was performed as previously described. After a preliminary cleaning step, our LAMP products were analysed by fluorescence spectroscopy recording emissions spectra in a 600 nm to 800 nm range with 630 nm excitation (Fig. 4A). All samples (Cy5 dilution series, no-Cy5 positive control, pure water and no enzyme control) indicated a sharp peak with maximum intensity at 630 nm and normalized fluorescence units (nFU) between 40.9 nFU to 76.5 nFU (scattered light peak). Additionally, only Cy5 containing LAMP samples showed a second, broader peak with maximum fluorescence values at 663–665 nm emission. Increasing the amount of Cy5 labelled nucleotides in the reaction mixture resulted in an increase of fluorescence intensities at 663 nm. There is no peak at 663 nm for ddH_2_O, no-Cy5 positive control and NEC.

Calculation of fluorophores measured in the purified LAMP amplicons was performed with the help of standard calibration line between 7.8E-03 mM and 2.5E-01 mM of free Cy5-dUTP (Fig. 4B). Using the values for the linear equation, calculation of the concentrations of incorporated, labelled nucleotides per LAMP reaction was possible. By that, the rate of Cy5-dUTP incorporation per 100 unlabelled dTTPs was determined as 0.05 by using 4.0E-03 mM Cy5d-dUTP per LAMP reaction and 1.70 with a ratio of 12.5% labelled dUTP/ 87.5% unlabelled dTTP. Furthermore, an average of 14.4% of all Cy5-dUTP used during LAMP was taken up by the *Bst3.0* polymerase, with no correlation to the Cy5-dUTP concentration per LAMP approach (Table 1).

**Table 1:** Yield of Cy5-dUTP incorporation.

Cy5-dUTP [mM]	Cy5-dUTP amount [%]	Theoretical per 100 nt	Direct Cy5 labelling		Indirect Cy5 labelling	
			Cy5-dUTP per 100 nt	Yield [%] Cy5-dUTP	AA-dUTP per 100 nt	Yield [%] AA-dUTP
4.0E−03	0.28	0.285	0.049	17.3	0.051	18.0
1.0E−02	0.71	0.709	0.097	13.7	–	–
2.0E−02	1.41	1.408	0.191	13.5	0.197	14.0
3.0E−02	2.10	2.098	0.359	17.1	–	–
4.0E−02	2.78	2.778	0.468	16.8	–	–
6.0E−02	4.11	4.110	0.586	14.2	–	–
8.0E−02	5.41	5.405	0.567	10.5	0.842	15.6
1.4E−01	9.09	9.091	1.119	12.3	–	–
2.0E−01	12.50	12.500	1.701	13.6	1.900	15.2

Depicted are the calculations for the x-dUTP incorporation during LAMP. First column shows the x-dUTP dilution series used in the different LAMP reactions followed by the column for the overall ratio of labelled dUTPs to unlabelled dTTPs in the reaction that is equal to the maximum resulting *theoretical* incorporation of labelled dUTP per 100 unlabelled dTTPs. The *labelling* columns showing the true incorporation rate per 100 dTTPs followed by the overall rate of incorporate x-dUTP out of all x-dUTP inserted in LAMP. Using direct labelling, labelling efficiency for large molecules like Cy5-dUTP were determined. Performing indirect labelling with subsequent fluorescent labelling, the incorporation yield using small molecules like AA-dUTP has been investigated (compare Fig. 1).

Additionally, LAMP products were analysed via standard gel electrophoresis using common SYBR green labelling (Fig. 4C) and the incorporated Cy5 molecules (Fig. 4D). In a SYBR green based visualization, all products amplified in an end point LAMP showed gel bands consistent in intensity and band pattern except NEC. Analysing the gel with Tecan LS reloaded microarray scanner (gel tray printed with 3D printing technology), different gel band and fluorescent intensities could be observed. While no-Cy5 positive control and NEC showed no signal, very weak fluorescent bands were seen in the samples with 4.0E-03 mM and 1.0E-02 mM Cy5-dUTP per LAMP reaction. By increasing the amount of labelled nucleotides for LAMP, the increased incorporation into amplicons became visible by increasingly brighter fluorescence bands.

In addition to the direct Cy5-dUTP labelling, the incorporation rate of small nucleotide modifications has been investigated. This was done by performing LAMP with AA-dUTP and subsequent Cy5-labelling described in the method section and Fig. 1. As before, the substitution rate was determined by fluorescence measurements. Using only 0.28% modified nucleotides, a substation rate (0.051 dUTP per 100 dTTP) comparable to the direct Cy5 labelling could be calculated. Increasing the AA-dUTP amount leads to slightly but not significantly higher rates. Thus, approximately 1.9 aminoallyl nucleotides out of 100 possible positions were incorporated when LAMP was performed with 2.0E-01 mM AA-dUTP. The mean overall yield of incorporated AA-dUTP of all available is 15.7%.

### Microarray analysis with pre-labelled LAMP amplicons

**Figure 5:**
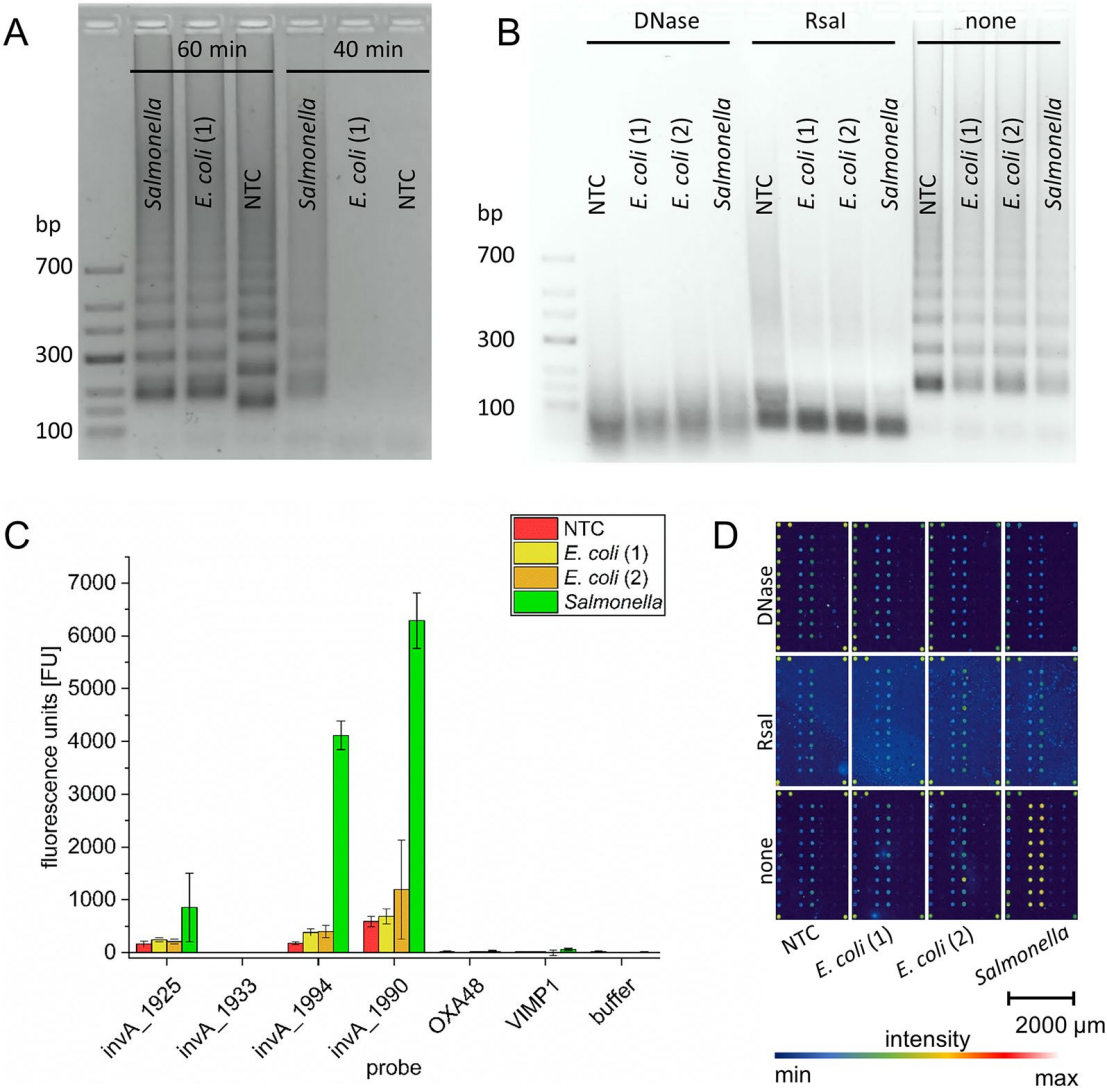
Microarray-based false-positive discrimination of Cy5-dUTP labelled *Salmonella* and *E. coli* samples. (**A**) A 60 min LAMP was performed to provoke false positive results. In addition, a 40 min LAMP was performed as a comparison. (**B**) Digestion with *DNAse* and *RsaI* produces smaller LAMP fragments. (**C**) Microarray analysis producing fluorescent signals after hybridization of digested and untreated *Salmonella* and control samples, is shown as graph and (**D**) false coloured display. The invA_x probes representing different *Salmonella* specific probes designed for the LAMP product. OXA48 and VIMP1 are unspecific control probes. (error bars depict standard deviation, n = 9).

The amplification time for our standard LAMP assay for *Salmonella spp.* is < 40 min, which is adequate for many applications. Nevertheless, when using 60 min to force false positive results, which is a common drawback of LAMP, false positives in the *E. coli spp.* control as well as in the no template controls (NTC) were observed (Fig. 5A). Cy5-dUTP LAMP and subsequent microarray analysis were combined to distinguish *Salmonella* amplicons and false positives. Prior to microarray hybridization, LAMP products were cleaved with an endonuclease and a restriction enzymes (*DNAse* and *Rsal*) and the products verified via gel electrophoresis (Fig. 5B). After 60 min LAMP with 6 ng genomic DNA per reaction, typical LAMP gel bands of none-cleaved products were detected in *Salmonella* as well as *E. coli* and NTC samples. By looking only at the band pattern in the gel, one cannot distinguish the different samples, except that NTC has a slightly different band pattern in some but not all cases.

The treatment of LAMP products with the specific enzyme (*RsaI*) and unspecific endonuclease (*DNase*) produced smaller fragments with a majority of less than 100 bp (Fig. 5B). For these products it was also not possible to distinguish between *Salmonella*, *E. coli* and NTC samples. After hybridising treated and untreated samples with *Salmonella*-specific probes, we could see clear differences in signal intensities in the false colour images (Fig. 5D). Enzyme treated and untreated controls (NTC and *E. coli*) as well as treated *Salmonella* samples showed fluorescence intensities only slightly brighter than the background. On the other hand, untreated *Salmonella* LAMP amplicons produced signal intensities clearly different from background and controls.

This could also be observed from the graphical evaluation (Fig. 5C). For the untreated Salmonella sample, the *InvA*-gen and LAMP sequence specific probes invA_1990 and invA_1994 showed signal intensities of more than 6000 FU and 4000 FU respectively. In addition, lower intensities of almost 1000 FU were detected for invA_1925, but no signals with invA_1933. Controls (*E. coli* and NTC) produced only 10%-19% signal intensities using invA_1990 and invA_1994 probes.

Control probes (OXA48 and VIMP1) as well as buffer spots showed no fluorescence.

## Discussion

Modifying LAMP products for a broad range of diverse applications is a commonly used technique and is regularly achieved by the incorporation of pre-modified primer sets.

Due to the complex primer set of up to six primers, the design and the evaluation of LAMP primers is not as simple as the design of PCR primers and can lead to false positive results if not carefully validated. Furthermore, in the methods described so far, the primers must be additionally prepared with appropriate molecules to enable their use, e.g. for LFA assays or spectroscopic analyses. In this study, we describe another promising method for labelling amplicons without using previously labelled primers.

Modifications of LAMP products by using dUTP instead of dTTP for usage in uracil DNA glycosylase (UDG-LAMP) assays have been shown previously^30,31^ indicating that LAMP enzymes like *Bst2.0* and *Bst3.0* are able to handle this nucleotide modification without causing serious amplification problems. Only a handful of publications are known in which additional modifications like digoxigenin-dUTP (DIG-dUTP)^32^ as well as fluorescent^29^ or biotin labelled nucleotides^23^ have been used.

In this study, various modified uracil nucleotides (x-dUTP) for the labelling of LAMP products were tested and validated. For this purpose, 5-(3-aminoallyl)-2'-deoxyuridine-5'-triphosphate (Cy5-dUTP), biotin-X-(5-aminoallyl)-dUTP (biotin-dUTP) and 5-aminoallyl-dUTP (AA-dUTP) represent different molecules with different sizes and properties. Cy5-dUTP allows direct labelling and the fluorescence signal can be used for amplicon detection directly after LAMP. Biotin-dUTP and AA-dUTP require at least one further reaction step. For example, Biotin-labelled amplicons can be used for immobilization via a biotin-streptavidin interaction in lateral flow assays (LFA). The aminoallyl-modification enables the downstream coupling of a wide range of other NHS-functionalised molecules for a broad spectrum of applications.

This study investigated whether the different modifications can be incorporated into the amplicons during the LAMP reaction. Furthermore, the effects on the LAMP reaction at different concentrations of the labelling molecules and the ratio of the incorporated labels were investigated.

To achieve this, two different labelling strategies were used. First, direct labelling with Cy5-dUTP and biotin-dUTP was performed without downstream reaction. Secondly, indirect labelling was carried out by incorporating functional groups (aminoallyl group) followed by chemical coupling of the target molecule (in this case NHS-Cy5).

Performing different detection methods such as qLAMP or spectroscopic and fluorescent gel analysis, it was not only possible to examine the influence of different molecule sizes but to show a broad range of downstream applications.

A commercially available LAMP kit, as described in the Methods and Materials section, was used for all experiments. To this, 7.5% DMSO was added as a preventive treatment to reduce false positives. This method has been described previously^33^ and showed the best results in our preliminary work. According to our knowledge, the DMSO has no influence on the labelling and was not explicitly added for this purpose.

This study shows that the introduction of different modifications for labelling LAMP products is possible. Small functional groups as well as larger molecules such as biotin and fluorescent dyes can be integrated into the amplicons. As shown in Fig. 2 and Fig. 3, amplification efficiency highly depends on the type of nucleotide modification, size and concentration. For the different modifications, a range from 0.28% x-dUTP/99.72% dTTP to 12.5% x-dUTP/87.5% dTTP was teste successfully.

When using large amounts of target DNA, in our example 4.2E + 00 ng of a 466 bp SARS-CoV-2 N-gene cDNA fragment, only Cy5-dUTP shows a slight impact of ΔCt = 2.96 min when using 12.5% Cy5-dUTP/87.5% dTTP compared to an unmodified amplification. The influence increases with the decrease of the target DNA. A 100-fold reduction to 4.2E-02 ng cDNA increased the ∆Ct to nearly 14 min using 12.5% Cy5 dUTP but only 30–60 s with 1.5% Cy5-dUTP. The effect of the Ct-value shift decreases also when using smaller nucleotide labels like biotin (4.4 min at 12.5%) or aminoallyl-group (2.9 min at 12.5%).

Cy5-dUTP-labelled LAMP products were successfully analysed by fluorescence spectrometry after amplification. It was shown that the fluorescence intensity of the amplicons increases with increasing concentration of the labelled nucleotides during LAMP. Fluorescence intensity increased 45-fold when the amount of Cy5-dUTP per LAMP reaction was increased from 4.0E-03 mM (0.28%) to 2.0E-01 mM (12.5%). On the basis of the data available, calculation of a Cy5-dUTP/dTTP substitution rate was possible of up to 1.7 per 100 dTTP when using 12.5% dUTP and about 0.2–0.5 per 100 dTTP when using 1.5%-2.8% dUTP during the LAMP reaction. Using only 0.3% fluorescence modified nucleotides, the labelling rate is less than 0.05 per 100 dTTP. Thus fluorescence intensity can be easily adjusted by using different amounts of labelling molecules during the LAMP process. Nevertheless, the average substitution rate of incorporate Cy5-dUTP out of all Cy5-dUTP available in LAMP is 14.4%.

It was also investigated whether the efficiency of labelling can be increased by using the indirect method. As shown in Fig. 1A, aminoallyl-modified dUTPs were incorporated into the amplicons for this purpose. Subsequently, the fluorescent dye NHS-Cy5 was coupled to the functionalised LAMP products and fluorescence intensity was measured as before. This showed that the use of smaller molecules (e.g. AA-dUTP compared to Cy5-dUTP) leads to better amplification efficiency, but does not seem to have a significant effect on labelling efficiency. The average total yield of incorporated AA-dUTP is 15.7% compared to 14.4% using Cy5-dUTP. This suggests that the activity of the LAMP enzyme is highly affected as a function of molecule size, but the rate of substitution is only affected slightly. Therefore, direct labelling is more reasonable as long as the labelling nucleotides with the desired modification are available. This eliminates post-processing steps such as coupling reactions and purification of the products. The only advantage of using smaller modifications is the speed of the LAMP assay as long as no subsequent coupling reaction is required. Nevertheless, the indirect method is a viable alternative if the desired modifications cannot be incorporated directly during LAMP.

In *Agarwal, S. *et al*., 2022* we used this fact to overcome problems with rapid SARS-CoV-2 detection. While fluorescein isothiocyanate labelling via FITC-dUTP in combination with a biotin-labelled primer seems to work for 30-min LAMP by *Zhang, C. *et al*. 2021*, we could not detect LAMP products after 10-min amplification time with our own assay. Changing the system to biotin-dUTP and FITC-labelled primer solved the problem^23^.

The use of 1.5% modified nucleotides under realistic assay conditions (Fig. 3B) resulted in only a marginal slowing of the amplification rate of less than 1 min when using Biotin-dUTP and AA-dUTP and just 3.5 min with Cy5-dUTP. In this case, 10E + 04 copies of SARS-CoV-2 cDNA fragments were used as starting material. This amount corresponds to the viral load in the sample of a mildly to moderately infected person^34^. These results show that sufficient labelling of amplicons can be achieved with both small molecules (AA-dUTP) and large fluorophores without significant loss of time. The use of less starting material (10E + 03 and 10E + 02 copies of cDNA) also resulted in successful amplification for all modifications used without false positives or false negatives. However, wider variations in Ct-values for both unlabelled and labelled amplicons made it impossible to determine the exact Ct-value shifts for this range (data not shown).

As a compromise between fluorescent intensity and amplification speed we recommend the use of only 1% to 2% labelled nucleotides when using large molecules like fluorophores and up to 5% with molecule sizes like biotin-dUTP.

Following this recommendation, it was possible to detect LAMP products not only via fluorescence spectroscopy and fluorescence gel electrophoresis but also by microarray analysis and 1.4% Cy5-dUTP per LAMP.

Microarray detection is a common and valuable tool for the analysis of PCR and isothermal amplification products^25,35,36^. Recently, we were able to present nucleotide-based labelling during recombinase polymerase amplification (RPA) followed by microarray as a very advantageous method^26^. In this study, we have successfully transferred this technique in a most intriguing way to detect and distinguish LAMP products.

*Salmonella* LAMP with 1.4% Cy5-dUTP and subsequent microarray detection was performed. As a proof of principle analysis this study investigated three main criteria. The first question related to the feasibility of detecting labelled amplicons using microarray technology in general, which was proved in this study. The second part was to investigate whether it is possible to discriminate false positive results. To this purpose, a 60-min LAMP was performed with false-positive no-template controls (NTC) and false-positive *E. coli* controls beside *Salmonella* samples. While the band pattern in the agarose gel (Fig. 5A) indicates different LAMP products in NTC and *E. coli* samples, no differences could be detected between *E. coli* and *Salmonella* samples. Using microarray technology and specific probes, we were finally able to distinguish the latter. The third aim was to investigate whether digestion of the LAMP products with restriction enzymes or nucleases is required to hybridize them successfully. For microarray detection, the usage of small DNA fragments is recommended to reduce difficulties in hybridization. Digestion of large DNA sequences can improve signal intensity^36,37^.

Because LAMP products can extend to very long lengths due to the amplification mechanism, *Salmonella*, *E. coli and* NTC amplicons were digested with nonspecific *DNase* and specific *RsaI* enzymes. While digestion of amplicons with both enzymes was detectable by gel electrophoresis, only untreated samples could be detected and distinguished by microarray. These results indicate that LAMP amplification can be combined directly with microarray detection to analyse and distinguish amplicons. Moreover, it can be performed without purification by simply adding hybridisation buffer after the LAMP reaction and incubation on an array. Thus, this application has the potential to be more specific than the commonly used lateral flow or colorimetric downstream detection methods. In this work, it was shown that the labelling of LAMP products using x-dUTP with 1–2% of labelled nucleotides produces excellent signals depending on the detection method used. We did not detect any significant loss of amplification or analytical performance compared to the previously published labelling methods. Further investigations on this, especially in direct comparison with primer-based labels, are currently the subject of further studies. However, results to date indicate that at least equivalent analytical performance is achieved with lateral flow-based, microarray-based and spectroscopic analyses. In addition, this method offers the possibility of optimising sensitivity by increasing the incorporation rates of labelling molecules, which is not possible when using single-labelled primers. In combination with suitable detection methods such as microarray technology, it should be possible to perform multiplex LAMP analyses without the need to use a large number of labelled primers.

In summary, this study shows that custom and flexible labelling of LAMP amplicons is possible using different modified nucleotides incorporated directly during the amplification process.

Thus, an easy-to-use and flexible alternative to the commonly used primer-based labelling was demonstrated, which is able to adjust the labelling efficiency more flexibly depending on the experimental question.

This allows easy adaptation to different downstream detection methods such as fluorescence spectroscopy, gel electrophoresis, microarrays, lateral flow systems or in-situ LAMP without the need for a pre-labelled primer.

## Methods and materials

### LAMP reaction and labelling reagents

25 µl LAMP with *Bst3.0* DNA Polymerase and reaction buffer (New England Biolabs; M0374S) was performed according to manufactures instructions (using a *Bio*-*Rad CFX* Real-Time PCR System) accept to the following changes: i) for all assays dimethyl sulfoxide (DMSO) was used with a final concentration of 7.5% (v/v) and the volume of DNA template per reaction was 1 µl; ii) for qLAMP assay 1 µl of 50 × SYBR green I (Invitrogen; S7563) was added; iii) aminoallyl-dUTP-XX-Cy5 (Jena Bioscience; NU-803-XX-CY5-L), biotin-11-dUTP (Jena Bioscience; NU-803-BIOX-L) and 5-aminoallyl-dUTP (Biutium; #40,020) were used for labelling of LAMP products with final concentrations ranging from 4.0E-03 mM to 2.0E-01 mM (0.28% to 12.5%) per reaction. The Amount of nuclease free water was adjusted to fit 25 µl reaction volume. DNA template concentration varied due to experimental settings (see results section). Primer and sample sequences are listed in Table 2.

**Table 2:** List of primer, probes and target sequences.

Template name	Template sequence		
SARS-CoV-2 cDNA (N-gene fragment) reference sequence: GenBank MN908947.3	GACCCCAAAATCAGCGAAATGCACCCCGC**A**TTACGTTTGGTGGACCCTCAGATTCAACTGGCAGTAACCAGAATGGAGAACGCAGTGGGGCGCGATCAAAACAACGTCGGCCCCAAGGTTTACCCAATAATACTGCGTCTTGGTTCACCGCTCTCACTCAACATGGCAAGGAAGACCTTAAATTCCCTCGAGGACAAGGCGTTCCAA**T**TAACACCAATAGCAGTCCAGATGACCAAATTGGCTACTACCGAAGAGCTACCAGACGAATTCGTGGTGGTGACGGTAAAATGAAAGATCTCAGTCCAAGATGGTATTTCTACTACCTAGGAACTGGGCCAGAAGCTGGACTTCCCTATGGTGCTAACAAAGACGGCATCATATGGGTTGCAACTGAGGGAGCCTTGAATACACCAAAAGATCACATTGGCACCCGCAATCCTGCTAACAATGCTGCAATCGTGCTACA		
*Salmonella spp*. (*invA* gene, only relevant section shown) reference sequence: GenBank M90846.1	GATATTGCCTACAAGCATGAAATGGCAGAACAGCGTCGTACTATTGAAAAGCTGTCTTAATTTAATATTAACAGGATACCTATAGTGCTGCTTTCTCTACTTAACAGTGCTCGTTTACGACCTGAATTACTGATTCTGgtacTAATGGTGATGATCATTTC**T**ATGTTCGTCATTCCATTACCTACCTATCTGGTTGATTTCCTGATCGCACTGAATATCgtacTGGCGATATTGGTGTTTATGGGGTCGTTCTACATTGACAGAATCCTCAGTTTTTCAACGTTTCCTGCGgtacTGTTAATTACCACGCTCTTTCGTCTGGCATTATCGAT**C**AgtacCAGTCGTCTTATCTTGATTGAAGCCGATGCCGGTGAAATTATCGCCACGTTCGGGCAATTCGTTATTGGCGATAGCCTGGCGGTGGG […]		

Target	Primer and sequence		Reference
SARS-CoV-2 cDNA (LAMP primer)	F3	CCCAAAATCAGCGAAATGCA	This study
	B3	AGCCAATTTGGTCATCTGGA	This study
	FIP	TGTTTTGATCGCGCCCCACTGATTACGTTTGGTGGACCCTC	This study
	BIP	TGCGTCTTGGTTCACCGCTCATTGGAACGCCTTGTCCTC	This study
	FL	TCCATTCTGGTTACTGCCAGTTGAA	This study
	BL	ACTCAACATGGCAAGGAAGACCTTA	This study
*Salmonella spp.* (LAMP primer)	F3	[TC]CTGGTACTAATGGTGATGA	Youn et al.^38^
	B3	[GCCTCG]ATCAAGATAAGACG	Youn et al.^38^
	FIP	AACACCAATATCGCCAGTACGATATGTTCGTCATTCCATTACCTAC	Youn et al.^38^
	BIP	TGACAGAATCCTCAGTTTTTCAACGATCGATAATGCCAGACGAAAG	Youn et al.^38^
	FL	GCGCGATCAGGAAATCAACC	Youn et al.^38^
	BL	CCTGCGGTATTGTTAATAACAACAC	Youn et al.^38^

Target	Microarray probes		Reference
*Salmonella spp.* (LAMP product)	invA_1900	*Linker-AGGTAGGTAATGGAATGACG	This study
	invA_1925	*Linker-GAACGACCCCATAAACACCA	This study
	invA_1933	*Linker-CCATAAACACCAATATCGCC	This study
	invA_1994	*Linker-AGGTAATGGAATGACGAACA	This study
*NC: E. coli* (OXA48-gene)**	OXA_48	*Linker-CGCTCCGATACGTGTAACTTA	This study
*NC: P. aeruginosa* (VIM-gene)**	VIM_P1	*Linker-GCGGAGATTGAAAAGCAAATTG	This study

LAMP sequences (F3 to B3 primer region) are underlined and bold letter mark the product from FIP to BIP primer. Complementary regions for microarray probes are bold underlined and recognition sequence for *RsaI* restriction enzyme is depicted in low case letters. For *Salmonella invA* gene, only the relevant sequence region is shown. *InvA* gene bases not shown are represented by […]. LAMP primer for *Salmonella spp.* were first published by Youn et al.^38^. B3/F3 bases in square brackets do not match our Salmonella sequence, but the LAMP still works very well. * amino-C6-T^13^; ** microarray negative control.

Depending on the experimental question, amplification times have been set to 20–60 min at 65 °C followed by an enzyme denaturation step of 5 min at 80 °C. For samples that were to be cleaved by restriction enzymes or nucleases, an additional heating step for 10 min at 90 °C was performed.

### LAMP post-preparation

LAMP products were purified with Mag-Bind® Total Pure NGS Kit (Omega Bio-Tek; M1378-01) according to the manufactures instructions and eluted in 30 µl ddH_2_O for subsequent gel electrophoretic and spectroscopic analysis. For spectroscopic analysis only, the fluorescently labelled DNA was purified by washing five times with 70% ethanol (v/v). Aminoallyl-products were purified twice. Once immediately after LAMP reaction to remove all amplification reagents (5 times ethanol wash) with a final elution step in 30 µl 0,1 M NaCO_3_-buffer (pH 8,4) as reaction medium for Cy5-NHS coupling. For this purpose, Cyanine-5 NHS-ester (Lumiprobe; 43,020) was pre-dissolved in DMSO and water was added to a final concentration of 10 mM. 2.5 µl was added to 22.5 µl LAMP product in NaCO_3_-buffer, mixed gently and incubated at 23 °C for 3 h in the dark. Afterwards the second purification step was performed with 10 times ethanol wash for complete removal of unbound fluorophores, ending with an elution step in 30 µl ddH_2_O.

Purification of microarray samples not treated with restriction enzymes was not performed.

Digestion of LAMP products using *RsaI* was performed by using 20 µl purified LAMP product (approx. 2 µg DNA), 2 µl enzyme (10 U/µl; New England Biolabs; R0167S) and 5 µl associated buffer (New England Biolabs; B6004S). Reaction mixture was filled up with ddH_2_O to final volume of 25 µl.

After digestion at 37 °C for 15 min, reaction was stopped by adding 10 µl of corresponding gel loading dye (New England Biolabs; B7024A).

Digestion of LAMP product using *RQ1 DNase* was performed by using 20 µl purified LAMP product (approx. 2 µg DNA), 1.6 µl enzyme (10 U/µl; Promega; M610A) , 4 µl associated reaction buffer (M198A) and 14.4 µl ddH_2_O. *DNase* digestion was carried out at 23 °C for 15 min with a final heat inactivation at 65 °C for 10 min.

### Templates and Primer

For the determination of labelling efficiency including the qLAMP, fluorescent spectroscopy and gel electrophoretic experiments a 466 bp cDNA fragment from SARS-CoV-2 N-gene (provided by Robert Koch Institute (RKI)) was used as well as LAMP primer designed with PrimerExplorer V5 (Table 2). Primers were designed based on the genome reference sequence MN908947.3 (GenBank, Table 2). The cDNA was amplified from the genomic RNA of the respective viruses in preparatory work. The extracted RNA was provided by the RKI.

Microarray analysis to distinguish *Salmonella spp.* and *E. coli* was performed by using 6 ng genomic DNA per reaction both. The genomic DNA of *Salmonella* and *E. coli* was extracted from overnight cultures using the DNeasy Blood & Tissue Kit (Quiagen; 69,504). *Salmonella* LAMP primer used in this study were first published by Youn et al.^38^.

Probes were designed and literature primer were compared based on the genome reference sequence M90846.1 available in GenBank (Table 2).

All primers were synthesized and provided by metabion AG.

### Gel electrophoresis

For gel electrophoretic analysis, 3 µl DNA Gel Loading Dye (6 x) (Thermo Fisher Scientific; R0611) were added to the purified (and digested) samples except the *RsaI* approach. For band visualization, a 2% agarose gel was prepared in 1 × TAE buffer (50 × TAE buffer; PanReac AppliChem; A1691) containing 4 µl peqGreen (VWR; 732-3196) per 100 ml gel. 12–15 µl of the purified and prepared samples were used for electrophoresis at 120 V for 1 h before detection via BioDoc Analyzer (Biometra) using a Canon EOS 1100D. GeneRuler Low Range DNA Ladder (Thermo Fisher Scientific; SM1191) and peqGOLD DNA ladder (VWR; 25-2040) were used for calculation of fragment length.

For the detection of incorporated Cy5-dUTP the DNA gel was scanned with a Tecan LS reloaded scanner using a 3D-printed gel tray.

### Fluorescent spectroscopy

The efficiency of fluorescence labeling with Cy5-dUTP was analysed with the PerkinElmer LS55 using FL Winlab software (PerkinElmer) and FLUOstar Omega (BMG Labtech). Using LS55 fluorescence spectrometer, purified LAMP products were diluted in a ratio of 1:16 (v/v) with a final volume of 96 µl and analysed using the following parameters: (i) 630 nm excitation and emission spectrum ranging from 600 to 800 nm; (ii) Emslit/Exslit = 10 nm; (iii) scan speed = 100 nm/min. All measurements were conducted as triplicates.

Using FLUOstar Omega, 30 µl purified samples were analysed without dilution using 620 nm excitation and 680 nm emission filter.

For the standard calibration line, free Cy5-dUTP was taken at a dilution range of 7.8E-03 mM to 2.5E-01 mM.

### Microarray analysis

Microarray analysis was performed using the hybridization protocol published in Warmt et al.^26^.

For this purpose, the corresponding probes were spotted onto 3D-epoxy glass slides (PolyAn GmbH) using a sciFLEXARRAYER SX (Scienion GmbH). After blocking with ethanolamine and subsequent washing steps, hybridization took place at 52 °C for 90 min. Subsequently, several washing steps were performed before the slide was analysed on the GenePix 4300 microarray scanner.

Probe sequences are listed in Table 2.

### Concentration measurements

Concentration measurements were conducted using Nanodrop 2000 (Thermo Fisher Scientific) with 1.5 µl purified LAMP products.

## Data Availability

The data that support the findings of this study are available from the corresponding author [C.W.] upon reasonable request. The reference sequences analysed and used for primer and probe design during the current study are available in GenBank under accession number MN908947.3 (SARS-CoV-2) and CP074639.1 (*Salmonella enterica*).
